# Treatment of Pruritus Ani Et Vulvæ

**Published:** 1848-11

**Authors:** 


*Treatment of Pruritus Ani et Vulvæ.*—M. Cazenave treats the above obstinate symptoms by one or other of the following lotions:

1. Subcarbonate of potass, - - - ʒ ij-ʒ iv.

   Distilled water, - - - - ℥ xvj.

2. Sulphuret of potass, - - - ʒ j.

   Distilled water, - - - - ℥ x.

3. Cyanide of potassium, - - - grs. ix.

   Distilled water, - - - - - ℥ viij.

4. Bichloride of mercury, - - - grs. iij-iv.

   Distilled water, - - - - - ℥ viij.

*Ranking's Abstract.*

